# A Two-Year Occurrence of *Fusarium* T-2 and HT-2 Toxin in Croatian Cereals Relative of the Regional Weather

**DOI:** 10.3390/toxins13010039

**Published:** 2021-01-07

**Authors:** Maja Kiš, Ana Vulić, Nina Kudumija, Bojan Šarkanj, Vesna Jaki Tkalec, Krunoslav Aladić, Mario Škrivanko, Sanja Furmeg, Jelka Pleadin

**Affiliations:** 1 Regional Veterinary Department Križevci, Croatian Veterinary Institute, Zakmandijeva 10, 48260 Križevci, Croatia; majakis72@gmail.com (M.K.); jaki.vzk@veinst.hr (V.J.T.); furmeg.vzk@veinst.hr (S.F.); 2 Laboratory for Analytical Chemistry, Croatian Veterinary Institute, Savska Cesta 143, 10000 Zagreb, Croatia; vulic@veinst.hr (A.V.); kudumija@veinst.hr (N.K.); 3 Department of Food Technology, University North, Trg dr. Žarka Dolinara 1, 43000 Koprivnica, Croatia; bojan.sarkanj@unin.hr; 4 Regional Veterinary Department Vinkovci, Croatian Veterinary Institute, Ul. Josipa Kozarca 24, 32100 Vinkovci, Croatia; k2aladic@gmail.com (K.A.); skrivanko@veinst.hr (M.Š.)

**Keywords:** *Fusarium* mycotoxins, T-2 toxin, HT-2 toxin, Weather, Occurrence, LC-MS/MS

## Abstract

To investigate into the T-2 and HT-2 toxin occurrence, 240 samples of unprocessed cereals (maize, wheat, barley, and oats) were sampled from different fields located in three Croatian regions during 2017–2018. In all samples, sum concentrations of T-2/HT-2 toxin were determined using the ELISA method, while the LC-MS/MS was used as a confirmatory method for both mycotoxins in positive samples (>LOD) and the establishment of T-2 over HT-2 toxin ratios. The results showed oats to be the most contaminated cereal, with T-2/HT-2 toxins detected in 70.0% of samples, followed by barley (40.9%), maize (26.8%) and wheat (19.2%), with the mean T-2/HT-2 ratio ranging from 1:2.7 in maize to 1:4.4 in oats. Sum T-2/HT-2 concentrations in two maize samples were higher than the indicative level recommended by the European Commission, necessitating subsequent investigations into the conditions under which these poorly investigated mycotoxins are produced. Statistically significantly (*p* < 0.05) higher concentrations of T-2/HT-2 toxin were determined in oats throughout study regions as compared to those found in wheat, but not maize and barley, while the concentrations of these mycotoxins were related to the regional weather in Croatia.

## 1. Introduction

Mycotoxin contamination of different cereal species and cereal-based products represents a ubiquitous food safety challenge, since these secondary fungal metabolites endanger human and animal health. Fungal species that mainly contaminate crops and produce mycotoxins belong to the *Fusarium*, the *Aspergillus* and the *Penicillium* genera [1]. Mycotoxin contamination may occur before harvest or during storage due to the inadequate storage conditions. Several factors contribute to the presence of mycotoxins in cereals, such as mechanical damage of kernels, pest infestation, mineral plant nutrition, poor harvest and storage practices and/or chemical treatment [2,3]. Furthermore, weather, such as air temperature and humidity, influence the colonisation of mycotoxigenic fungi and mycotoxin production [4,5,6].

Both T-2 and HT-2 toxin belong to the group of trichothecene mycotoxins synthesized by various *Fusarium* species. *Fusarium sporotrichioides,* as the major producer of T-2 and HT-2 toxin, grows within the temperature range spanning from −2 °C to 35 °C, best with water activities above 0.88 [1,7]; therefore, it can be frequently found on grains coming from temperate regions. Water activity and temperature optimal for the biosynthesis of T-2 and HT-2 are 0.980–0.995 and 20–30 °C, respectively [8,9]. Due to the broad presence of T-2 and HT-2-producing fungi and conditions favouring T-2 and HT-2 biosynthesis, contamination with these mycotoxins may occur in different grains [10,11] and goes in favour of widespread toxin occurrence on a global scale. 

It has been established that T-2 toxin has pronounced cytotoxic, immunosuppressive and haematotoxic effects, and can cause chronic human and animal disorders [12]. Since T-2 toxin is quickly metabolized to HT-2 toxin after ingestion, the two are considered to be equally toxic. The major effect of T-2 toxin is the inhibition of protein synthesis, but also (at higher doses) the inhibition of RNA and DNA synthesis and lipid peroxidation, as well as apoptosis [13,14]. T-2 toxin primarily affects the haematopoietic and the immune system, inducing changes in proliferation and differentiation of red blood cells and leukocyte count, depressed antibody formation, and lymphoid cell depletion [1,12]. Due to the lack of valid sub-chronic or chronic toxicity studies and based on the limited evidence on carcinogenicity in experimental animals, the International Agency for Research on Cancer (the IARC) classified T-2 toxin into the Group 3 of agents not classifiable as human carcinogens [15]. 

Data on the occurrence of T-2 and HT-2 toxin in feed and food across Europe have shown considerable temporal and geographical variations [16,17,18]. In addition to agrotechnical factors affecting mould formation and mycotoxin synthesis, mycotoxin levels are known to depend on local weather, in particular air temperature and precipitation, which can markedly affect mould colonization of cereals, causing the production of mycotoxins to vary across climate zones worldwide [19]. Due to the scarce data on the occurrence of T-2 and HT-2 toxins in cereals and cereal-based products, the European Commission (EC) recommended the member states to collect evidence-based data on annual variations of these mycotoxins in order to establish their maximal permissible levels (MPLs) [10]. The Commission Recommendation 2013/165/EU provided indicative levels for the sum concentrations of T-2 and HT-2 in cereals and cereal-based products, above which investigations of the factors leading to the presence of both mycotoxins should be performed. The EC also recommended further studying of factors responsible for relatively high levels of T-2 and HT-2 toxin, in order to develop containment and prevention strategies [20]. 

Given that weather impact on the occurrence of toxicogenic moulds and mycotoxins has been recognized all over the world, and bearing in mind that contamination of cereals with *Fusarium* mycotoxins has often been seen in Croatia lately [21,22], the objective of this study was to explore into the occurrence of T-2 and HT-2 toxin in unprocessed Croatian cereals. Additionally, the aim of the study was to gather data on the influence of weather recorded during the two-year study period on the production of these mycotoxins in cereal samples taken from the fields located in three main cereal production regions in Croatia. 

## 2. Results and Discussion 

### 2.1. Analytical Methods’ Validation and Quality Control

The values obtained by the certified reference material (CRM) analysis, run for the sake of the ELISA method quality control, ranged from 82.7 μg/kg to 96.5 µg/kg for T-2 toxin and from 83.6 µg/kg to 94.1 µg/kg for HT-2 toxin, and were within the assigned values given by the CRM manufacturer. 

As presented in the earlier study by Pleadin et al. [23] the limits of detection (LODs) and the limits of quantification (LOQs) of the implemented ELISA method varied dependent of the analysed material and amounted to 9.1 μg/kg and 14.6 μg/kg in maize (respectively), 14.5 μg/kg and 20.1 μg/kg in wheat (respectively), 24.6 μg/kg and 29.8 μg/kg in barley (respectively), and 16.2 μg/kg and 20.7 μg/kg in oat (respectively). Table 1 summarises the results of validation and quality control, obtained with pseudo-blank oats and CRM, respectively, descriptive of the LC-MS/MS.

The LC-MS/MS method was fully validated with the LOD values spanning from 1.2 to 1.6 ng/g and LOQs spanning from 4.3 μg/kg to 5.3 μg/kg. The trueness, obtained by analysing the certified reference material of the oat flour sample, were: 91.5% of the mean certified value for T-2 toxin and 88.7% of the mean certified value for HT-2 toxin. Both analytes had satisfactory linearity results (correlation coefficients, 0.998 for T-2 and 0.999 for HT-2 toxin). The recovery expressed against the values assigned to T-2 toxin ranged from 77.0% to 102.6%, while those expressed against the values assigned to HT-2 toxin spanned from 84.9% to 101.5%. The average recovery values for both mycotoxins are presented in Table 1. Relative standard deviations for both mycotoxins were below 10%, which is satisfactory, although the method can be further improved by implementing an internal standard. In that case, the quantification would be more accurate. 

Based on the obtained validation and quality control results, the applied methods were considered suitable for the reliable determination of sum T-2/HT-2 concentrations (ELISA) and their individual (LC-MS/MS) concentrations in unprocessed cereals, and were in the line with the criteria given under Commission Decision 2002/657/EC [24].

### 2.2. The Occurrence of T-2 and HT-2 Toxin in Cereals

Sum T-2/HT-2 toxin concentrations in raw maize, wheat, barley and oat, obtained in this study during 2017 and 2018 using the ELISA method, are presented in Table 2. The presence of T-2/HT-2 toxin was detected in 33.8% of the analysed samples. The study results show a statistically significantly higher (*p* < 0.05) representation of T-2/HT-2 toxin in oats (70.0%) as compared to other cereals. The highest mean sum of T-2/HT-2 toxin concentration was ascertained in oats (87.9 ± 63.1 µg/kg), followed by maize (54.1 ± 85.5 µg/kg) and wheat (23.0 ± 9.0 µg/kg), while the lowest sum concentration of these toxins was found in barley (22.6 ± 11.8 µg/kg). In two maize samples, sum concentrations of T-2/HT-2 toxin were higher (332.3 µg/kg and 252.8 µg/kg, respectively) than the indicative level stipulated for maize (200 µg/kg). In the case of repetitive findings, these results deserve further research into the matter. Although the highest mean T-2/HT-2 concentration was detected in oats, concentrations of these toxins found in the investigated small grain cereals were generally below the indicative levels [20].

The presence of T-2 and HT-2 toxin as natural contaminants of different feedstuffs and foodstuffs, strongly depends on many parameters of influence, among which the most important are the type and the resistance of cereals, and weather during flowering and harvesting time [5]. Together with cereals, cereal-based products are the main source of human and animal exposure to T-2 and HT-2 toxin; therefore, newly-established tolerable intake and acute reference dose values for T-2/HT-2 toxin [25] mirror a rising concern about food and feed safety.

The available occurrence data for T-2/HT-2 mycotoxin in cereals mostly report either only T-2 toxin concentration or sum T-2/HT-2 concentrations, with limited data on HT-2 toxin concentration alone. A global survey indicated that European cereals were mainly contaminated by *Fusarium* mycotoxins deoxynivalenol, zearalenone and T-2 toxin, which are most often co-occurring due the same producer [26]. However, T-2 toxin levels reported in European crop food and kindred products vary greatly, depending on the type of cereal, country of origin, local weather, and else, with the maximum concentrations of up to 2406 µg/kg found in naturally-contaminated oats [10,23,26].

Several studies on T-2 toxin occurrence in Europe suggest that oat is the cereal most frequently contaminated with T-2 toxin [11,27], which is confirmed with the results obtained in this study. Schoneberg et al. [17] reported annual variations (65–76%) in T-2/HT-2 toxin contamination of Swiss oats during 2013–2015. The highest quantified concentrations of T-2/HT-2 toxins in their study were 1091 µg/kg and 3789 µg/kg, respectively [17]. Italian malting barley samples had the percentage of positives ranging from 22% to 53% during 2011–2014 harvesting seasons, with sum T-2/HT-2 concentrations ranging between 26 µg/kg and 787 µg/kg [16]. The incidence of T-2/HT-2 toxin in maize samples from Serbia was 53.3%, with the maximum sum concentration of 209.0 µg/kg [28]. Di Marco Pisciottano et al. [29] detected a widespread contamination with both T-2 and HT-2 toxin in Italian cereals during the 2015–2019 harvesting seasons. In their study, oat and barley were particularly contaminated, and an even higher contamination was determined in compound feeds.

Previous Croatian research showed T-2 toxin contamination of 24.4% maize samples, with the highest concentration of 210 µg/kg, indicating a *Fusarium* contamination of maize after heavy rainy periods seen during 2010 [30]. In the study targeted at the crops harvested in 2011, maize, wheat, barley and oat samples had the highest prevalence of T-2 toxin (57%, 25%, 32% and 18% of the tested cereals contaminated, respectively) [21]. In another study by Pleadin et al. [22], oat was the most contaminated cereal of them all. The mean sum concentration of T-2/HT-2 toxin was found in oats (136 ± 55.6 µg/kg), followed by maize (94.8 ± 63.7 µg/kg), wheat (65.6 ± 25.2 µg/kg) and barley (61.3 ± 20.6 µg/kg), which is comparable to the results obtained in this study. The authors also reported the maximal T-2 toxin concentration of 128 µg/kg and the maximal HT-2 toxin concentration of 256 µg/kg, both found in a maize sample [22].

A well-established LC-MS/MS confirmatory method was implemented for the assessment and confirmation of T-2 and HT-2 concentrations. The confirmation was performed only in cereals in which the sum T-2/HT-2 concentrations surpassed the ELISA’s LOD (Table S1). T-2 and HT-2 toxin were found to co-occur in the contaminated cereals, HT-2 toxin thereby accounting for roughly 2/3 of the sum T-2/HT-2 toxin concentration. The average T-2 over HT-2 toxin ratios ranged from 1:2.7 in maize to 1:4.4 in oats (Table 3), which is comparable to other literature sources [21,27,31]. Higher T-2/HT-2 toxin ratios found in some oat samples (up to 1:11.0) may be the result of T-2 toxin hydrolysis and its transformation into degradable metabolites [27]. In this study, maize was the cereal most contaminated with T-2 and HT-2 toxin, with the highest concentrations of 128.5 µg/kg and 224.4 µg/kg, respectively.

When comparing the sum T-2 and HT-2 toxin concentrations obtained by the LC-MS/MS to the sum T-2/HT-2 toxin concentrations determined by the ELISA, a slight difference in the two can be seen in terms of mainly lower figures obtained by the LC-MS/MS method (Table S1). This difference can be explained by the proneness of ELISA method to unwanted cross-reactions with conjugated metabolites, resulting in an increased metric uncertainty [10], lower specificity and higher cross-reactivity of the method.

### 2.3. Regional Weather and T-2/HT-2 Toxin Concentrations in Each Production Year

**Figure 1:**
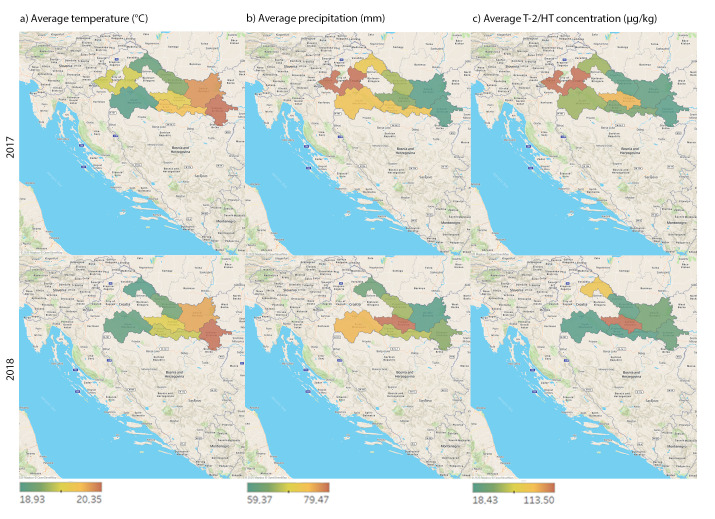
Maps representing regional weather and sum T-2/HT-2 toxin concentrations obtained in unprocessed cereals in three Croatian cereal-producing regions during 2017 and 2018 (May–October): (**a**) average temperatures (°C); (**b**) average precipitation (mm); (**c**) mean sum T-2/HT-2 toxin concentrations (positive samples, >LOD of the ELISA method).

The climate of continental parts of Croatia is moderate. However, climate changes consequent to the global warming trend are also witnessed in Croatia in the last decades, with weather significantly deviating from its long-term patterns. The Official National Weather Reports for 2017 and 2018 showed extremely hot weather during cereal growth and harvest seasons (May–October) all over Croatia. The maps representing the average temperatures and precipitation in Croatia during 2017–2018 and the mean T-2/HT-2 toxin concentrations determined in unprocessed cereals across three different Croatian regions, are shown in Figure 1a–c.

Weather witnessed in 2017 in the investigated parts of Croatia during the final phenological stages (May–July) of small grain cereals’ growth, can be described as extremely hot, with precipitation in line with the seasonal average. During July and August (the anthesis and the silking period), the region in which the highest level of T-2/HT-2 toxin was found in maize, i.e., eastern Croatia, was characterized by extremely high average air temperatures (mean, 24 °C) and sparing quantities of rainfall (between 30 mm and 60 mm). Drought, as the strongest abiotic factor that puts a plant in the state of stress, has a direct effect on colonization of moulds of the *Fusarium* genus [32]. In the subsequent course, abundant rainfall was recorded during September and October 2017 in all investigated regions, so that ripening continued in a high humidity environment, which could also have contributed to the colonization of *Fusarium* moulds and, consequently, to higher T-2/HT-2 toxin contamination. Contrary to the hot weather with drought-stress recorded in 2017, precipitation during 2018 was in line with the long-term average, with a slightly more abundant precipitation recorded in the eastern region. Higher precipitation combined with an extremely warm weather witnessed during 2018, resulted in conditions favouring the growth of *Fusarium* moulds and the occurrence of T-2/HT-2 toxins, especially in the northern and the eastern region.

During the period of cereal growth and harvesting, the average temperature in 2017 was ranging from 18.2 °C to 19.7 °C, and in 2018 from 18.9 °C to 20.4 °C, while the highest average temperature in both years was observed in two sub-regions of the region 1 (Vukovar-Srijem district coloured red and Osijek-Baranja district coloured orange in Figure 1a). In spite of the fact that these regions were shown to have the highest average temperature, their responsibility for the highest T-2/HT-2 toxin concentrations failed to be proven. Bearing in mind that the temperature range that favours the growth of *Fusarium* moulds and consequently also T-2/HT-2 toxin biosynthesis is 20–30 °C [1,7,8,9], and that the average temperature measured during 2017 and 2018 was below or slightly above the lower limit, a significant impact of temperature on T-2/HT-2 toxin biosynthesis could not be expected. Unlike the environmental temperature-toxin concentration relationship, the relationship between the average precipitation level and high concentrations of T-2/HT-2 toxin was established. During 2017, the highest average precipitation was found in region 2—northern Croatia (Koprivnica-Križevci district coloured red in Figure 1b,c) and region 3—central Croatia (Zagrebačka district, also coloured red in Figure 1b,c). During 2017, the highest concentrations of T-2/HT-2 toxin were found exactly in these regions. During 2018, the relationship between the highest average precipitation and the highest concentrations of T-2/HT-2 toxin was established as well, but only in region 1 (Požega-Slavonija district coloured red Figure 1b,c). These results suggest a possible influence of precipitation on T-2/HT-2 toxin concentrations and its responsibility for the peak toxin values found in cereals coming from the above regions.

In this study, the highest T-2/HT-2 toxin concentration (332.3 µg/kg, i.e., above the indicative level) was detected in maize sampled from northern Croatia during 2018. Weather across this region, seen during maize development stages (anthesis, silking, fruit development, ripening), can be described as extremely warm, with medium to high precipitation (between 40–90 mm). Furthermore, in one maize sample collected from the eastern region during 2017, the detected sum T-2/HT-2 toxin concentration was 252.8 µg/kg (above the indicative level stipulated for maize), which suggests that high moisture during maize ripening influences water activity and consequently the development of moulds and T-2/HT-2 toxins. However, further research should be done to identify other agronomic factors, such as previous crop, cultivation, host cultivar and fungicide application [33], which could also have influenced the *Fusarium* mycotoxins’ occurrence in the harvested cereals.

**Figure 2:**
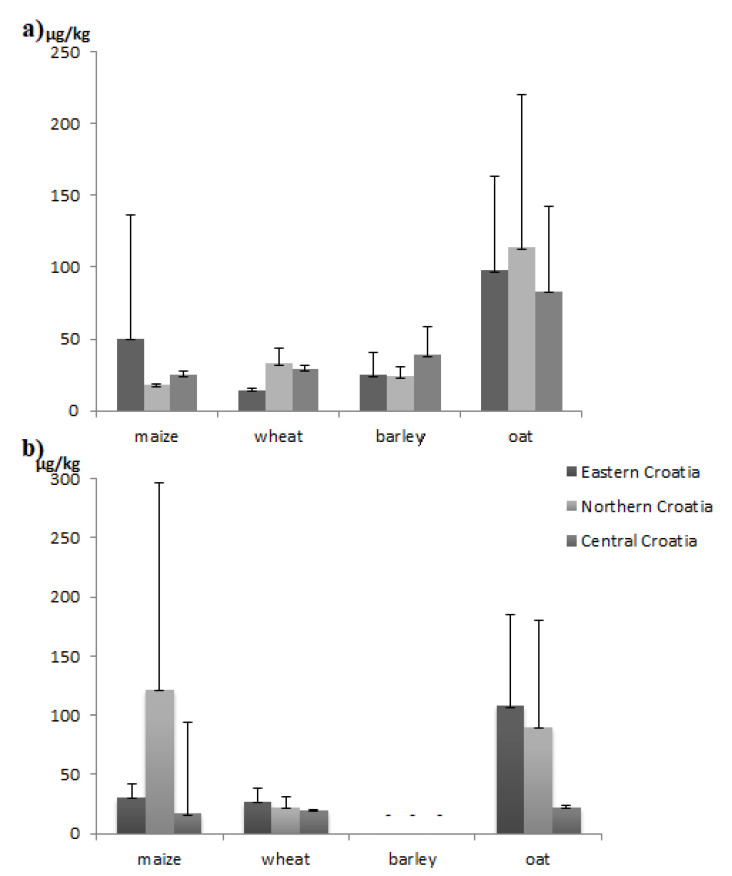
Mean sum (±SD) T-2/HT-2 toxin concentrations in unprocessed cereals sampled across three different Croatian regions during (**a**) 2017; (**b**) 2018. Mean concentration found in positive samples (>LOD of the ELISA method); Short horizontal line (-) was used when no positive samples (>LOD of the ELISA method) of a given type of cereal (in this case barley harvested in 2018) were detected.

Mean sum T-2/HT-2 toxin concentrations obtained in unprocessed cereals in each of the three Croatian regions during 2017 and 2018 are shown in Figure 2a,b).

Statistical analysis showed that sum T-2/HT-2 toxin concentrations did not statistically significantly vary between the production years (2017 and 2018 (*p* = 0.292). The same goes for the comparison between the level of mycotoxin contamination (sum concentrations of these mycotoxins across the three production regions, i.e., Eastern, Northern and Central Croatia) (*p* = 0.322). However, a statistically significant difference (*p* < 0.001) in the sum T-2/HT-2 toxin concentrations was found in different types of cereals, oats thereby being the most contaminated as compared to the other three. When comparing the sum T-2/HT-2 toxin concentrations found in each type of cereal under study in each study region and each production year, a marked difference was found only for wheat (*p* = 0.033), not maize (*p* = 0.325) and oats (*p* = 0.769).

Although T-2/HT-2 concentration determined in most of the sampled cereals was significantly lower than the stipulated indicative levels, higher concentrations of T-2 and HT-2 toxin determined in two maize samples can be linked to substantial temperature variations and high precipitation seen during maize growth and harvesting period. However, due to the interaction of various factors that may affect the biosynthesis of T-2 and HT-2 toxin during cereal cultivation, such as mechanical damage of kernels, pest infestation, mineral plant nutrition, poor harvest and storage practices, and/or chemical treatment [2,3], the observed high concentrations cannot be ascribed solely to the weather.

The results of this study conform to other studies from countries with climate similar to Croatia. In the Romanian study performed by Stanciu et al. [34], it was concluded that drought periods during grain formation or high moisture in the late preharvest period favour HT-2 toxin formation in wheat. Kos et al. [35] showed that high precipitation recorded during maize growing season had a considerable impact on the synthesis of *Fusarium* mycotoxins in Serbian maize. Data published earlier in Croatia also demonstrated that certain weather conditions favour the growth of *Fusarium* moulds and that contamination of cereals with *Fusarium* mycotoxins is possible, especially during rainy and warmer periods [3,21,22].

## 3. Conclusions

The present study contributes to better understanding of the influence of regional weather on the occurrence of T-2/HT-2 toxin in cereal samples grown on Croatian fields and intended to be used by households and industries in Croatia. The results showed the highest occurrence of T-2/HT-2 toxin in oats, followed by barley, maize and wheat. With an exception of two maize samples, the levels of T-2/HT-2 toxins ascertained in the analysed cereal samples were lower than the indicative levels recommended by the European Commission. Significantly higher concentrations of T-2/HT-2 toxin were determined in oats throughout study regions as compared to those found in wheat, but not maize and barley, while the concentrations of these mycotoxins were related to the regional weather in Croatia. Yet, further studies are needed in order to identify measures to be taken during cultivation and storage to prevent T-2/HT-2 contamination of cereals. Given the explicit toxicity of T-2 and HT-2 toxin, their synergistic effects and high incidence in cereals, it is necessary to systematically monitor these mycotoxins in all stages of food and feed production, as well as to stipulate their maximum permitted amounts in different types of foodstuffs and feedstuffs.

## 4. Materials and Methods

### 4.1. Samples

A total of 240 samples of unprocessed cereal species (71 maize, 73 wheat, 66 barley and 30 oat samples) were collected from May to October in both 2017 and 2018. The samples were cultivated on, and sampled from, different fields in three sampling regions in continental Croatia (region 1—Eastern Croatia embracing Brod-Posavina, Vukovar-Srijem, Osijek-Baranja, Virovitica-Podravina and Požega-Slavonija districts, *n* = 150 samples; region 2—Northern Croatia, in specific Koprivnica-Križevci district, *n* = 85 samples; region 3—Central Croatia embracing Sisak-Moslavina and Zagrebačka district, *n* = 5 samples). Samples were obtained directly from farmers or medium-size domestic enterprises (additional information on cereal species unknown to us) and were intended to be used by domestic industries. Prior to the analysis, the samples were not treated in any manner. Sampling and sample preparation were performed in line with the provisions of Commission Regulation No. 401/2006 [36], stipulating the methods of sampling to be exercised within the frame of monitoring of mycotoxin levels in food. The aggregate samples of raw cereals were combined of three incremental samples weighing at least 1 kg.

Samples were transported to the analytical site within 72 h and stored in a cool and dry environment. The prepared test portions (1 kg per sample) were ground into a fine powder (at least 300 g of the sample) using an analytical mill (Cyclotec 1093, Tecator, Sweden) with a 1.0-mm sieve. Samples were kept in polyethylene bags and stored at 4 °C until analysis was carried out within the next 48–72 h.

### 4.2. Chemicals, Standards and Reagents

All chemicals used for ELISA and LC-MS/MS analysis (acetic acid, acetonitrile and methanol purchased from Honeywell, Offenbach, Germany) were of a HPLC grade. T-2 and HT-2 toxin standards (both 100 µg/mL in acetonitrile) were supplied by Sigma-Aldrich Chemie GmbH (Steinham, Germany). A Ridascreen T-2/HT-2 toxin kit used with the ELISA method was provided by R-Biopharm (Darmstadt, Germany). PuriTox Total Myco-MS solid phase clean-up columns used within the frame of the LC-MS/MS method were supplied by R-BiopharmRone LTD (Glasgow, Scotland). The certified reference material (CRM) of the oat flour was purchased from Fapas, Fera Science Ltd. (York, UK). The assigned reference values were 85.3 ± 13.7 µg/kg for T-2 toxin and 86.9 ± 11.9 µg/kg for HT-2 toxin.

### 4.3. Determination of MYCOTOXINs

Sum T-2/HT-2 toxin concentrations were first determined using a competitive ELISA test kit according to the instructions provided by the kit manufacturer. Briefly, the extraction of ground samples (5 g) was carried out with 25 mL of methanol/distilled water solution (70/30; *v*/*v*) for maize, wheat and barley samples, or, for oat samples, with 25 mL of the appropriate extraction buffer provided in the ELISA test kit. The obtained filtrate was diluted in 1:2 ratio and pipetted into the ELISA test kit. The ELISA tests were evaluated using a ChemWell auto-analyser (Awareness Technology Inc. 2910, Palm City, FL, USA), with the absorbance set at 450 nm. Sum T-2/HT-2 concentration in an individual sample was recalculated based on the calibration curve, and multiplied by the used dilution factor, thereby duly respecting the average recovery values.

Further LC-MS/MS analysis was conducted only for samples in which the established T-2/HT-2 concentrations were above the ELISA’s respective limit of detection (LOD). To that end, 2.5 g of a test portion were extracted with 10 mL of 80%-acetonitrile, blended for 10 min on a head-over shaker and then centrifuged (10 min, 4000 rpm at room temperature). To 2 mL of the obtained supernatant, 20 µL of acetic acid were added. Exactly 1.4 mL of the acidified extract was passed through the PuriTox Total Myco-MS column. A part of the obtained filtrate (0.5 mL) was diluted with 1500 µL of 1%-acetic acid. To the diluted sample solution, 100 µL of 1%-acetic acid in 20% acetonitrile were added, vortexed, and then 20 µL of it injected directly into the LC-MS/MS system.

The LC-MS/MS system was used as described by Kiš et al. [37]. The analytes were separated on a Poroshell 120 EC-C18 (particle size 2.7 µm, dimensions 3.0 × 50 mm) (Agilent Technologies, Santa Clara, CA, USA). Gradient elution was employed with a mobile phase consisting of 0.1%-formic acid (eluent A) and methanol (Eluent B) at the flow rate of 500 µL/min and the temperature of 40 °C. The following gradient was applied: 0–0.5 min 80% A, 4.0 min 60% A, 8.0 min 5.0% A, 10.1 min 80% A. MS/MS conditions were as follows: positive electrospray ionization (ESI+) mode, ion source temperature 350 °C, drying gas flow 9 L/min, nebulizer 45 psi, and the capillary voltage 6 kV. Table 4 shows the exact values descriptive of the ions monitored during the LC-MS/MS analysis.

### 4.4. Validation and Quality Control of the Analytical Methods

The implemented ELISA method was validated earlier in the research by Pleadin et al. [23]. For this study, quality control of the ELISA method was performed by analysing the CRM in parallel with each analysis of the studied samples, to check whether the obtained sum T-2/HT-2 toxin concentrations fall within the assigned range. The LOD and the LOQ of the LC-MS/MS method were estimated according to the Guidance document of merit [38] via paired observations. T-2 and HT-2 standard solutions were used for spiking pseudo-blank oat samples at the level of 10 μg/kg. After analysis, the difference in signal abundances of pseudo-blank and spiked samples was used for LOD and LOQ calculation. Linearity was confirmed in the range of 0.5–50 ng/mL, using mixed and diluted standards of T-2 and HT-2 toxins, while the trueness was tested using six standard CRM replicates and subsequent comparing to the values assigned for each mycotoxin. The recovery was tested with each sample batch, also using oat flour as the CRM. The determination of these validation parameters was also described by Kiš et al. [37].

### 4.5. Meteorological Data

Data on weather conditions in Croatia during cereal growth and harvest season (–October 2017 and 2018) were obtained by accessing the Croatian Meteorological and Hydrological Service’s official website (https://meteo.hr). Based on the data on the average monthly air temperatures and precipitation, obtained at more than 30 weather stations closest to the individual cereal growth micro-locations, maps were designed using the Google Fusion Tables (Google, Mountain View, CA, USA).

### 4.6. Statistical Analyses

Statistical analysis utilised the Statistica Ver. 10.0 Software (StatSoft Inc. 1984–2011, Tulsa, OK, USA) and the analysis of variance (ANOVA plus the Tamhane’s T2 post hoc test), with the statistical significance set at 95% (*p* = 0.05). To determine the statistical significance of sum T-2/HT-2 toxin concentrations, the Mann–Whitney U and the Kruskal–Wallis H tests were performed.

## Figures and Tables

**Table 1:** The results of validation and quality control of the LC-MS/MS method employed for the determination of T-2 and HT-2 toxin in oats.

Validation ^1^/Quality Control ^2^ Parameter	Mycotoxin	
	T-2 Toxin	HT-2 Toxin
LOD ^1^ (μg/kg)	1.2	1.6
LOQ ^1^ (μg/kg)	4.3	5.3
Correlation coefficient ^1^ (linearity)	0.998	0.999
Trueness ^1^ (%)	91.5	88.7
Average recovery ^2^ (% ± RSD ^a^)	85.3 ± 9.6	93.9 ± 5.8

^a^ Relative standard deviation. ^1^ Validation parameters. ^2^ Quality control parameters.

**Table 2:** Sum concentrations of T-2/HT-2 toxin in raw cereals determined by the ELISA method and the samples with concentrations higher than the limit of detection and the indicative levels.

Cereals	IL ^a^ (µg/kg)	Positive ^b^ (%)	Higher than IL ^c^	Mean ^d^ (µg/kg)	SD (µg/kg)	Min (µg/kg)	Max (µg/kg)
Maize (*n* = 71)	200	26.8	2	54.1	85.5	15.6	332.3
Wheat (*n* = 73)	100	19.2	0	23.0	9.0	12.2	42.1
Barley (*n* = 66)	200	40.9	0	22.6	11.8	12.2	52.1
Oat (*n* = 30)	1000	70.0	0	87.9	63.1	14.3	212.8

^a^ Indicative levels (IL) of sum concentrations of T-2/HT-2 toxin, above which further research should be done in case of repetition [19]. ^b^ The percentage of samples in which sum T-2/HT-2 concentrations were higher than the limit of detection (>LOD). ^c^ The number of samples in which sum T-2/HT-2 concentrations were higher than the indicative levels. ^d^ Mean concentration found in positives (>LOD).

**Table 3:** Descriptive statistics of T-2 and HT-2 toxin concentrations determined in ELISA-positive raw cereals using the LC-MS/MS.

	T-2 Toxin (µg/kg)				HT-2 Toxin (µg/kg)				Share T-2:HT-2 ^a^
Cereals	Mean	SD	Min	Max	Mean	SD	Min	Max	
Maize (*n* = 7)	40.5	53.5	7.4	128.5	69.7	77.3	20.1	224.4	1:2.7
Wheat (*n* = 4)	8.7	2.3	6.4	11.7	25.5	4.4	20.1	30.4	1:3.0
Barley (*n* = 5)	10.8	3.3	6.7	15.9	32.7	8.9	20.7	42.1	1:3.1
Oat (*n* = 17)	23.6	16.9	5.3	59.0	80.2	47.8	20.2	160.5	1:4.4

^a^ The ratio of mean T-2 over mean HT-2 toxin concentration.

**Table 4:** Ions monitored within the frame of the LC-MS/MS analyses targeted at T-2 and HT-2 toxin determination [37].

Mycotoxin	Precursorion	Fragmentor Voltage (V)	Productions	Collision Energy (eV)	Cell Accelerator Voltage (V)
T-2 toxin	489.2	200	387.1 ^b^	20	1
			245.1 ^a^	27	
HT-2 toxin	447.2	100	345.1 ^a^	18	1
			285.1 ^b^	20	

^a^ The best signal-to-noise ratio used as the quantifier; ^b^ The second best signal-to-noise ratio used as the qualifier.

## Data Availability

Data are available upon request, please contact the contributing authors.

## Supplementary Materials

The following are available online at https://www.mdpi.com/2072-6651/13/1/39/s1, Table S1. Concentrations of T-2 and HT-2 toxin determined by both ELISA and LC-MS/MS method in unprocessed cereals in which sum concentrations of these toxins surpassed the ELISA’s limit of detection (LOD), shown for each cereal species, year of sampling and the production district seated in one of the three Croatian regions under study.
