# Supplementary material for: A Two-Year Occurrence of Fusarium T-2 and HT-2 Toxin in Croatian Cereals Relative of the Regional Weather
Source: Toxins (Basel). 2021 Jan 7;13(1):39. doi: 10.3390/toxins13010039 (PMC7827321; doi:10.3390/toxins13010039)
Supplement: Supplementary file 1 [file toxins-13-00039-s001.pdf]

A Two-Year Occurrence of *Fusarium* T-2 and HT-2 Toxin in Croatian Cereals Relative of the Regional Weather

Maja Kiš, Ana Vulić, Nina Kudumija, Bojan Šarkanj, Vesna Jaki Tkalec, Krunoslav Aladić, Mario Škrivanko, Sanja Furmeg and Jelka Pleadin

Table S1. Concentrations of T-2 and HT-2 toxin determined by both ELISA and LC-MS/MS method in unprocessed cereals in which sum concentrations of these toxins surpassed the ELISA's limit of detection (LOD), shown for each cereal species, year of sampling and the production district seated in one of the three Croatian regions under study.

Cereals Species	Year of Sampling	District of Croatia	Concentrations (µg/kg)			
			ELISA Sum T-2/HT-2	LC-MS/MS Sum T-2/HT-2	LC-MS/MS T-2	LC-MS/MS HT-2
Maize (n = 7)	2017	Požega-Slavonija	277.5	252.8	128.5	124.3
	2017	Brod-Posavina	47.3	45.1	12.3	32.8
	2017	Brod-Posavina	30.4	28.4	8.3	20.1
	2017	Osijek-Baranja	38.9	35.2	11.5	23.7
	2018	Koprivnica-Križevci	48.7	42.2	7.4	34.8
	2018	Osijek-Baranja	41	35.6	7.9	27.7
	2018	Osijek-Baranja	323.5	332.3	107.9	224.4
Wheat (n = 4)	2017	Koprivnica-Križevci	45.6	42.1	11.7	30.4
	2018	Koprivnica-Križevci	32.1	27.5	7.4	20.1
	2018	Vukovar-Srijem	30.2	30.5	6.4	24.1
	2018	Vukovar-Srijem	40.5	36.5	9.3	27.2
Barley (n = 5)	2017	Brod-Posavina	60.3	52.1	10.2	42.1
	2017	Osijek-Baranja	50.2	40.9	10.4	30.5
	2017	Vukovar-Srijem	49.5	45.1	15.9	29.2
	2017	Koprivnica-Križevci	32.5	27.4	6.7	20.7
	2017	Koprivnica-Križevci	54.9	51.8	10.6	41.2
Oat (n = 17)	2017	Požega-Slavonija	65.8	59.3	9.4	49.9
	2017	Požega-Slavonija	60.3	58.2	13.9	44.3
	2017	Požega-Slavonija	67.5	65.2	7.5	57.7
	2017	Požega-Slavonija	80.4	70.3	11.6	58.7
	2017	Požega-Slavonija	214.8	203.8	44.5	159.3
	2017	Koprivnica-Križevci	87.9	90.2	22.9	67.3
	2017	Koprivnica-Križevci	230.4	212.8	52.3	160.5
	2017	Zagrebačka	125.1	90.3	11.5	78.8
	2018	Koprivnica-Križevci	200.2	179.1	22.68	156.42
	2018	Koprivnica-Križevci	86.4	67	5.4	61.56
	2018	Koprivnica-Križevci	91.6	72.4	25.2	47.16
	2018	Koprivnica-Križevci	73.1	112.3	32.04	80.28
	2018	Koprivnica-Križevci	264.4	192.4	35.46	156.96
	2018	Koprivnica-Križevci	187.2	121	59.04	61.92
	2018	Požega-Slavonija	30.8	25.4	5.3	20.1
	2018	Požega-Slavonija	30.4	30.7	8.6	22.1
	2018	Požega-Slavonija	129.3	114.5	34.3	80.2

Region 1—Eastern Croatia: Brod-Posavina, Vukovar-Srijem, Osijek-Baranja, Virovitica-Podravina and Požega-Slavonija district

Region 2—Northern Croatia: Koprivnica-Križevci district

Region 3—Central Croatia: Sisak-Moslavina and Zagrebačka district
